# *De Novo* Purine Metabolism is a Metabolic Vulnerability of Cancers with Low p16 Expression

**DOI:** 10.1158/2767-9764.CRC-23-0450

**Published:** 2024-05-02

**Authors:** Naveen Kumar Tangudu, Raquel Buj, Hui Wang, Jiefei Wang, Aidan R. Cole, Apoorva Uboveja, Richard Fang, Amandine Amalric, Baixue Yang, Adam Chatoff, Claudia V. Crispim, Peter Sajjakulnukit, Maureen A. Lyons, Kristine Cooper, Nadine Hempel, Costas A. Lyssiotis, Uma R. Chandran, Nathaniel W. Snyder, Katherine M. Aird

**Affiliations:** 1 Department of Pharmacology and Chemical Biology and UPMC Hillman Cancer Center, University of Pittsburgh School of Medicine, Pittsburgh, Pennsylvania.; 2 Department of Biomedical Informatics and UPMC Hillman Cancer Center, University of Pittsburgh School of Medicine, Pittsburgh, Pennsylvania.; 3 Tsinghua University School of Medicine, Beijing, P.R. China.; 4 Department of Cardiovascular Sciences, Aging + Cardiovascular Discovery Center, Lewis Katz School of Medicine, Temple University, Philadelphia, Pennsylvania.; 5 Department of Molecular and Integrative Physiology, Department of Internal Medicine, Division of Gastroenterology, and Rogel Cancer Center, University of Michigan, Ann Arbor, Michigan.; 6 Genomics Facility, UPMC Hillman Cancer Center, University of Pittsburgh School of Medicine, Pittsburgh, Pennsylvania.; 7 Biostatistics Facility, UPMC Hillman Cancer Center, University of Pittsburgh, Pittsburgh, Pennsylvania.; 8 Division of Hematology/Oncology, Department of Medicine, UPMC Hillman Cancer Center, University of Pittsburgh, Pittsburgh, Pennsylvania.

## Abstract

p16 is a tumor suppressor encoded by the *CDKN2A* gene whose expression is lost in approximately 50% of all human cancers. In its canonical role, p16 inhibits the G_1_–S-phase cell cycle progression through suppression of cyclin-dependent kinases. Interestingly, p16 also has roles in metabolic reprogramming, and we previously published that loss of p16 promotes nucleotide synthesis via the pentose phosphate pathway. However, the broader impact of p16/*CDKN2A* loss on other nucleotide metabolic pathways and potential therapeutic targets remains unexplored. Using CRISPR knockout libraries in isogenic human and mouse melanoma cell lines, we determined several nucleotide metabolism genes essential for the survival of cells with loss of p16/*CDKN2A*. Consistently, many of these genes are upregulated in melanoma cells with p16 knockdown or endogenously low *CDKN2A* expression. We determined that cells with low p16/*CDKN2A* expression are sensitive to multiple inhibitors of *de novo* purine synthesis, including antifolates. Finally, tumors with p16 knockdown were more sensitive to the antifolate methotrexate *in vivo* than control tumors. Together, our data provide evidence to reevaluate the utility of these drugs in patients with p16/*CDKN2A*^low^ tumors as loss of p16/*CDKN2A* may provide a therapeutic window for these agents.

**Significance:**

Antimetabolites were the first chemotherapies, yet many have failed in the clinic due to toxicity and poor patient selection. Our data suggest that p16 loss provides a therapeutic window to kill cancer cells with widely-used antifolates with relatively little toxicity.

## Introduction

p16, encoded by the *CDKN2A* gene, is a critical cell cycle regulator that inhibits CDK4/6 (1). Given its importance in the cell cycle, p16 is a well-known tumor suppressor with approximately 50% of all human tumors showing decreased expression or activity of p16 through a variety of mechanisms, including hypermethylation or deletion of the *CDKN2A* locus or mutations (2–4). While the *CDKN2A* gene encodes both p16 and p14, cancer-associated mutations are more commonly found in the p16 open reading frame (5, 6), suggesting a more critical regulatory role of p16 in cancer. In melanoma, approximately 30% of patients have a deep deletion of the *CDKN2A* locus and 10%–15% have *CDKN2A* mutations (7). In spite of this high prevalence, there are currently no FDA-approved therapies specifically tailored for these patients. However, ongoing clinical trials are exploring the efficacy of CDK4/6 inhibitors in combination with both mutant BRAF inhibitors and immune checkpoint blockade (refs. 8, 9; clinicaltrials.gov).

We and others have published that p16 has noncanonical roles beyond cell cycle control, including metabolic reprogramming (10–12). We previously found that loss of p16 expression promotes nucleotide synthesis in part via increased translation of the pentose phosphate pathway (PPP) enzyme ribose-5-phosphate isomerase A (RPIA; ref. 12). However, glucose tracing through the PPP indicated that additional pathways may contribute to the increase in steady-state nucleotide levels observed in these cells. As there is currently no available RPIA inhibitor, our study aimed to identify additional targetable nucleotide metabolism pathways that are specifically vulnerable to inhibition in cancers with low p16/*CDKN2A* expression (p16/*CDKN2A*^low^). We used melanoma as a model because approximately 40% of melanomas have decreased p16, typically due to 9p21 chromosomal loss (7).

Nucleotide homeostasis is critical for cellular fidelity serving various functions including energy production, biomass synthesis, signaling, and DNA repair (13–16). As such, *de novo* nucleotide synthesis relies on multiple metabolic pathways, and nucleotides can also be salvaged through catabolism or the microenvironment (17–20). Glucose via the PPP is used to synthesize phosphoribosyl pyrophosphate, the pentose sugar backbone of all nucleotides. One carbon metabolism, essential for *de novo* purine synthesis, relies on serine metabolism though the folate cycle (21). In addition, metabolites such as aspartate, glycine, and glutamine further contribute to *de novo* nucleotide synthesis, connecting a variety of metabolic pathways with nucleotide biosynthesis (20). Although cancer cells often exhibit upregulated nucleotide biosynthesis pathways, and historically antimetabolites including antifolates have been used as chemotherapy, their potential efficacy in targeting p16/*CDKN2A*^low^ tumors remains widely underexplored.

Using CRISPR knockout (KO) screens in human and mouse melanoma isogenic pairs with knockdown of p16 and *Cdkn2a*, respectively, alongside data mining of the Dependency Map (DepMap), we identified 31 common nucleotide metabolism genes negatively enriched in p16/*CDKN2A*^low^ cells. Many of these genes are either transcriptionally or translationally upregulated in p16/*CDKN2A*^low^ cells. Therapeutic targeting of pathways that promote nucleotide synthesis, including antifolates, demonstrated that purine but not pyrimidine biosynthesis is a metabolic vulnerability of p16/*CDKN2A*^low^ cells *in vitro*. Consistently, treatment of mice bearing shp16 tumors with the antifolate methotrexate led to decreased tumor burden without associated toxicity. Importantly, treatment with antifolates had a more robust long-term inhibitory effect compared with the CDK4/6 inhibitor palbociclib. Finally, mining of publicly available data showed that patients with melanoma with genetic alterations in enzymes associated with *de novo* purine synthesis and one carbon metabolism exhibit significantly worst overall survival. Together, our data suggest that p16/*CDKN2A* loss may create a therapeutic window to kill cancer cells with widely-used antifolates with minimal toxicity.

## Materials and Methods

### Cell Lines

SKMEL28 (RRID:CVCL_D4WR) and Yumm5.2 (RRID:CVCL_JK43) cells were purchased from ATCC and used within 30 passages. ATCC performs cell line authentication by short tandem repeat profiling. Cells were used within 6 months of receipt or resuscitation. SKMEL28 cells were cultured in DMEM (Thermo Fisher Scientific, catalog no. MT10013CV) supplemented with 5% FBS (BioWest, catalog no. S1620). Yumm5.2 cells were cultured in DMEM and Ham's F-12 50/50 media (Thermo Fisher Scientific, catalog no. MT10092CV) supplemented with 10% FBS (BioWest, catalog no. S1620) and nonessential amino acids (Corning, catalog no. 25-025-Cl). Both were supplemented with 1% Penicillin/Streptomycin (Thermo Fisher Scientific, catalog no. 15-140-122). All cell lines were tested monthly for *Mycoplasma* as described in ref. 22.

### Lentiviral Packaging and Infection

The human short hairpin RNA (shRNA) hairpin pLKO.1-shp16 (TRCN0000010482), the murine shRNA hairpins pLKO.1-sh*Cdkn2a* #1 (TRCN0000077816) and #2 (TRCN0000362595) and the human and murine pLKO.1-shGFP control (Addgene, catalog no. 30323; RRID:Addgene_30323) vectors were packaged using the ViraPower Kit (Invitrogen, catalog no. K497500) following the manufacturer's instructions. Cells were infected with corresponding vectors for 16 hours and selected for 3 days with 1 µg/mL of puromycin (SKMEL28) or 3 µg/mL of puromycin (Yumm5.2).

### Design of *Cdkn2a* CRISPR KO Murine Cell Line

Single-stranded oligonucleotides targeting mouse *Cdkn2a* (forward: CACCGGCTGGATGTGCGCGATGCC and reverse: AAACGGCATCGCGCACATCCAGCC, Integrated DNA Technologies) or intergenic region control (forward: CACCGAGTGTTCCTAGAGATAGAAG and reverse: AAACCTTCTATCTCTCTAGGAACACTC) were annealed, phosphorylated, and ligated into pLentiCRISPRv2 (Addgene, catalog no. 52961; RRID:Addgene_52961), kindly gifted by Feng Zhang. Lentivirus was packaged using the ViraPower Kit as described above and used to infect Yumm5.2 cells. Mixed pooled population Yumm5.2 cells infected with intergenic region control single-guide RNAs (sgRNA) were used as control while a single clone was obtained for Yumm5.2 cells targeted with *Cdkn2a* sgRNAs.

### Nucleotide Metabolic CRISPR Library Construction

We engineered a pooled sgRNA library containing 2,700 sgRNAs targeting various genes either directly or indirectly related to nucleotide metabolism along with 315 controls targeting intragenic regions as described previously (23). Briefly, we used publicly available CRISPR sgRNA design tools that optimize on-target and minimize off-target genome editing (http://crispr.dfci.harvard.edu/SSC/) and pooled human metabolic library (24) to identify 10 sgRNAs for each gene. The pooled oligo library was synthesized by Twist Bioscience. The oligo library was cloned into lentiCRISPRv2 (Addgene, catalog no. 52961; RRID:Addgene_52961) as described previously (23). Briefly, target gRNAs library was PCR amplified using NEB Next High-Fidelity PCR Master Mix (New England Biolabs, catalog no. M0541S) and purified (Qiagen, catalog no. 28104) following the manufacturer's instructions. The 140 bp target gRNA library was separated in a 2% agarose gel and gel extracted (Qiagen, catalog no. 28704). The lentiCRISPRv2 backbone was digested with Esp3I (Thermo Fisher Scientific, catalog no. FERFD0454), separated in a 1% agarose gel, and gel extracted (Qiagen, catalog no. 28704) following the manufacturer's instructions. Purified target gRNA library and digested backbone were assembled in a Gibson assembly reaction (New England Biolabs, catalog no. E2611L), and isopropanol precipitated using GlycoBlue Coprecipitant (Invitrogen, catalog no. AM9515) following the manufacturer's instructions. To ensure optimal sgRNA representation, the library was sequenced, obtaining a coverage of >95%.

### CRISPR KO-based Screens

The human nucleotide-focused CRISPR KO library containing 128 genes comprising the nucleotide metabolism signature (Supplementary Table S1), was designed as stated above. The mouse metabolic-focused CRISPR KO library was a gift from Dr. Kivanc Birsoy (Addgene, catalog no. 160129; RRID:Addgene_160129). The screening using the human nucleotide-focused library was conducted on SKMEL28 shGFP control and shp16 cells, while the screening using the mouse metabolic-focused library was conducted on Yumm5.2 shGFP control and sh*Cdkn2a* #1 cells. Briefly, the appropriate number of cells were infected with pooled libraries at an multiplicity of infection (MOI) < 0.3 to achieve >400-fold library coverage after selection. Selection was conducted with 1 µg/mL of puromycin (SKMEL28) or 3 µg/mL puromycin (Yumm5.2) for 6 days. Cells were passed every 2 days and the whole population was seeded to maintain the library coverage throughout. After selection, cells were harvested for genomic DNA extraction using the Zymo Research kit (catalog no. D4069). sgRNA inserts were PCR amplified using Ex Taq DNA Polymerase (Takara, catalog no. RR001A) from sufficient genome equivalents of DNA to achieve an average coverage of >200x of the sgRNA library. See primers in Supplementary Table S2. Pooled PCR amplicons per sample were then sequenced. MAGeCK was used as the bioinformatics pipeline to analyze negatively and positively enriched genes (25). Briefly, read counts from the samples were median-normalized to adjust for library size effect and read count distribution and mean-variance modeling was used to capture the relationship of mean and variance. Next, negative binomial (NB) model was used to assess whether there are significant differences in sgRNA abundance between shp16 and shcontrol groups similarly to as the method used for differential expression analysis in bulk RNA sequencing (RNA-seq; ref. 26). *P* values are then calculated from the NB model and used to rank sgRNAs using an α-RRA algorithm (27) to identify positively or negatively selected genes. α-RRA operates under the assumption that if a gene has no impact on cell survival, target it using a sgRNA should be evenly distributed in the ranked list of all sgRNAs. Therefore, the algorithm ranks genes by comparing the skew in rankings with a uniform null model, prioritizing genes with consistently higher or lower-than-expected sgRNA rankings. The statistical significance of the skew is determined through permutation as described in ref. 25. Supplementary Tables S3 and S4 contain the gene names (Gene), Pathway, *P*-values (*P*-value), log_2_-fold change (shp16/*Cdkn2a* vs. shcontrol, lfc), and scores (score) for the human and mouse CRISPR KO screen, respectively. Because the mouse CRISPR library focuses on the whole metabolism, we have selected only those genes that belong to nucleotide metabolism. Because nucleotide metabolism is a small pathway and the goal was to determine the relative necessity of these 128 genes for the proliferation/survival of shp16/sh*Cdkn2a* cells, genes were considered “negatively enriched” with a log_2_FC < 0 and “positively enriched” with a log_2_FC > 0. While intergenic controls were included in both libraries and use for the bioinformatics analysis, they have been removed from the graphs and tables.

### Western Blotting

Cells lysates were collected in 1X sample buffer (2% SDS, 10% glycerol, 0.01% bromophenol blue, 62.5 mmol/L Tris, pH 6.8, 0.1 mol/L DTT) and boiled to 95°C for 10 minutes. Protein concentration was determined using the Bradford assay (Bio-Rad, catalog no. 5000006). An equal amount of total protein was resolved using SDS-PAGE gels and transferred to nitrocellulose membranes (Cytiva, catalog no. 10600001) at 110 mA for 2 hours at 4°C. Membranes were blocked with 5% nonfat milk in TBS containing 0.1% Tween-20 (TBS-T) for 1 hour at room temperature. Membranes were incubated overnight at 4°C in primary antibodies diluted in 4% BSA/TBS + 0.025% sodium azide. Membranes were washed four times in TBS-T for 5 minutes at room temperature after which they were incubated with horseradish peroxidase (HRP)-conjugated secondary antibodies for 1 hour at room temperature. After washing four times in TBS-T for 5 minutes at room temperature, proteins were visualized on film after incubation with SuperSignal West Pico PLUS Chemiluminescent Substrate (Thermo Fisher Scientific, catalog no. 34580). Primary antibodies were: Rabbit anti-*Cdkn2a* (Abcam, catalog no. ab108349; RRID:AB_10858268; 1:1,000); Rabbit anti-p16 INK4A (Cell Signaling Technology, catalog no. 29271; 1:1,000); mouse anti-retinoblastoma protein (Rb; Thermo Fisher Scientific, catalog no. MA5-11387; RRID:AB_10987096; 1:500), mouse anti-β-actin (Sigma-Aldrich, catalog no. A1978; RRID:AB_476692; 1:10,000). Secondary antibodies were: Anti-mouse IgG, HRP-linked (Cell Signaling Technology, catalog no. 7076; RRID:AB_330924; 1:10,000), Anti-Rabbit IgG, HRP-linked (Cell Signaling Technology, catalog no. 7074; RRID:AB_2099233; 1:5,000).

### Metabolite Analysis by Liquid Chromatography-High Resolution Mass Spectrometry

Metabolites were measured by hydrophobic interaction liquid chromatography-high resolution mass spectrometry after sample extraction by methanol/water precipitation adapted from previously published approaches (28). For quantification, samples were quenched in 1 mL prechilled −80°C 80/20 methanol:water (v/v) and spiked with 50 µL of 1 µmol/L isotope-labeled tricarboxylic acid cycle mix (Cambridge Isotope Laboratories, catalog no. MSK-TCA-A) prediluted in 80/20 methanol:water. For isotope tracing, the internal standard was omitted. Samples were vortexed for 1 minute, returned to −80°C for 30 minutes, centrifuged at 18,000 × *g* 10 minutes at 4°C, and the supernatant was transferred to a deep well 96-well plate and evaporated to dryness under nitrogen gas. Samples were reconstituted in 100 and 2 µL of the sample was injected from a 4°C autosampler onto a 25°C ZIC-pHILIC 150 × 2.1 mm 5 µm particle size column (EMD Millipore) with a ZIC-pHILIC 20 × 2.1 guard column in a Vanquish Duo UHPLC System (Thermo Fisher Scientific). Chromatography conditions were: buffer A acetonitrile; buffer B 20 mmol/L ammonium carbonate, 0.1% (v/v) ammonium hydroxide in water without pH adjustment, with a gradient of 0.5 minutes at 20% A then a linear gradient from 20% to 80% B; 20–20.5 minutes: from 80% to 20% B; 20.5–28 minutes: hold at 20% B at a 0.150 mL/minute flow rate. Elute was introduced to a Q Exactive Plus with a HESI II probe operating in polarity switching mode with full scans from 70 to 1,000 m/z with an insource fragmentation energy of 1. Instruments were controlled via XCalibur 4.1, and data were analyzed on Tracefinder 5.1 using a 5 ppm window from the predominant hydrogen-loss negative ion. Area under the curve (AUCs) for each analyte was normalized to the matched internal standard or the nearest surrogate internal standard.

### Proteomics

SKMEL28 cells were homogenized in 50 mmol/L TEAB, 5% SDS. Total protein was measured by Micro BCA™ Protein Assay Kit (Pierce, catalog no. 23235). Five-hundred micrograms of total protein was digested on S-TRAP MIDI columns (Protifi, catalog no. NC1679156) per manufacture protocol and desalted on Peptide Desalting Spin Columns (Pierce, catalog no. 89852). Phosphopeptides were enriched on an AssayMAP Bravo (Agilent) with Fe^3+^ column. Liquid chromatography-trapped ion mobility spectrometry-tandem mass spectrometry (LC-TIMS-MS/MS) analysis was carried out using a nanoElute UHPLC system (Bruker Daltonics) coupled to the timsTOF Pro mass spectrometer (Bruker Daltonics), using a CaptiveSpray nanoelectrospray (Bruker Daltonics). Roughly 100 ng of peptide digest or phosphopeptide enrichment was loaded on a capillary C18 column (25 cm length, 75 µm inner diameter, 1.6 µm particle size, 120 Å pore size; IonOpticks). Peptides were separated at 55°C using a 60-minute gradient at a flow rate of 300 nL/minute [mobile phase A: 0.1% formic acid (FA); mobile phase B (MPB): 0.1% FA in acetonitrile]. A linear gradient of 2%–35% MPB was applied for 60 minutes, followed by a 5-minute wash at 95% MFB before equilibrating the column at 2% MFB for 6 minutes. The timsTOF Pro was operated in PASEF mode collecting full-scan mass spectra from 100 and 1,700 m/z. Ion mobility resolution was set to 0.60–1.60 V·s/cm over a ramp time of 100 ms. Data-dependent acquisition was performed using 10 PASEF MS-MS scans per 1.1 second cycle. Active exclusion time window was set to 0.4 minutes, and the intensity threshold for MS-MS fragmentation was set to 2.5e^4^ while low m/z and singly charged ions were excluded from PASEF precursor selection. MS-MS spectra were acquired via ramped collision energy as function of ion mobility.

The LC/MS-MS data were analyzed with the MaxQuant software suite (version 2.1.3; ref. 29). The Andromeda protein identification search engine (30) and a SwissProt human protein database (downloaded on November 11, 2022 with 20,403 entries) were utilized with default settings for Orbitrap instruments. The parameters used included a precursor mass tolerance of 20 ppm for the first search and 4.5 ppm for the main search, a product ion mass tolerance of 0.2 Da, and a minimum peptide length of 5 amino acids. Trypsin was set as the proteolytic enzyme with a maximum of two missed cleavages allowed. The enzyme specifically cleaves peptide bonds C-terminal of arginine and lysine if they are not followed by proline. Carbamidomethylation of cysteine was set as a fixed modification. Oxidation of methionine, deamination of both asparagine and glutamine, and acetylation of the protein N-terminus were set as variable modification. Phosphorylation of serine, threonine, and tyrosine was set as a variable modification for phosphopeptides. A 1% FDR was used to filter the peptide identification results. The integrated feature intensities provide a relative measure of abundance for each feature at the peptide level and used in all subsequent analyses.

### Proliferation Assays

An equal number of cells were seeded in multiwell plates and cultured for 4–5 days. Proliferation was assessed by fixing the cells for 5 minutes with 1% paraformaldehyde after which they were stained with 0.05% crystal violet. Wells were destained using 10% acetic acid. Absorbance (590 nm) was measured using a spectrophotometer (BioTek Epoch Microplate reader).

### RNA Isolation, Sequencing, and Analysis

Total RNA was extracted from cells with TRIzol (Ambion, catalog no. 15596018) and DNase treated, cleaned, and concentrated using Zymo columns (Zymo Research, catalog no. R1013) following manufacturer's instructions. RNA integrity number (RIN) was measured using BioAnalyzer (Agilent Technologies; RRID:SCR_019715) RNA 6000 Nano Kit to confirm RIN above 7 for each sample. The cDNA libraries, next-generation sequencing, and bioinformatics analysis was performed by Novogene. Raw and processed RNA-seq data can be found on Gene Expression Omnibus (GEO; GSE243717; RRID:SCR_005012).

### Polysome Profiling and Sequencing

Eight culture plates per condition (∼23 million cells per condition) were incubated with harringtonine (2 µg/mL) for 2 minutes at 37°C followed by 5 minutes of cycloheximide (100 µg/mL) treatment at 37°C. Cells were washed twice with PBS after each treatment. Cells were scraped in 600 µL of lysis buffer (50 mmol/L HEPES, 75 mmol/L KCl, 5 mmol/L MgCl_2_, 250 mmol/L sucrose, 0.1 mg/mL cycloheximide, 2 mmol/L DTT, 1% Triton X-100 and 1.3% sodium deoxycholate and 5 µL of RNase OUT) on ice. Lysates were rocked for 10 minutes at 4°C and centrifuged at 3,000 × *g* for 15 minutes at 4°C. A total of 400 µL of lysates supernatant (cytosolic cell extracts) were layered over cold sucrose gradients (10 mmol/L HEPES, 75 mmol/L KCl, 5 mmol/L MgCl_2_, 0.5 mmol/L EDTA and increasing sucrose concentrations from 20% to 47%). Gradients were centrifuged at 34,000 rpms in a Beckman SW41 rotor for 2 hours and 40 minutes at 4°C. After centrifugation, low (0 to 2 ribosomes) and high (>2 ribosomes) polysome fractions were collected in TRIzol (1:1) using a density gradient fractionation system (Brandel) equipped with a UA-6 absorbance detector and a R1 fraction collector. RNA was DNase treated, cleaned, and concentrated using Zymo columns (Zymo Research, catalog no. R1013). The cDNA libraries, next-generation sequencing, and bioinformatics analysis was performed by Novogene. Raw and processed polysome fractionation followed by RNA-seq (Poly-seq) data can be found on GEO (GSE243717; RRID:SCR_005012).

### DepMap Data

For analysis of dependency scores, CRISPR (DepMap Public 23Q2+Score, Chronos) was downloaded for cutaneous melanoma cell lines (July 2023). Cells were characterized as *CDKN2A* high or low based on mRNA expression (Expression Public 23Q2). Cell lines with *CDKN2A* mutations were excluded. A similar method was used to analyze proteomics and drug sensitivity using the PRISM Repurposing Primary Screen.

### IncuCyte Cytotoxicity Assay

Live cell viability was assessed using IncuCyte S3 imaging system (Sartorius). Briefly, an equal number of cells per condition were seeded in triplicates in a 96-well plate and treated with, methotrexate (Cayman Chemical Company, catalog no. 13960) or lometrexol hydrate (Cayman Chemical Company, catalog no. 18049), in the presence of the highly sensitive cyanine nucleic acid dye 1x green Cytotox reagent (Sartorius, catalog no. ESS4633) for SKMEL28 cells or 1x red Cytotox reagent (Sartorius, ESS4632) for Yumm5.2 cells. Cells were imaged for 3 days with live cell imaging every 3 hours. Dead cell quantification was performed using the IncuCyte software.

### Annexin V/Propidium Iodide Staining and Flow Cytometry

For Annexin V/7AAD experiments, SKMEL28 cells in triplicates were treated for 5 days with 0.17 µmol/L methotrexate or 0.12 µmol/L lometrexol in DMEM + 5% FBS, while Yumm5.2 cells in triplicate were treated for 3 days with 22 nmol/L of methotrexate or 13 nmol/L of aminopterin. Cells were then stained with Annexin V (Thermo Fisher Scientific, catalog no. R37176) in 2.5 mmol/L Ca^2+^ containing DMEM for 15 minutes at room temperature. Prior to FACS analysis, 40 ng of propidium iodide was added to the cell suspension (eBiosciences, catalog no. 00-6990-50). Data were acquired in LSRFortessa cell analyzer (BD Biosciences) and analyzed using FlowJo software (RRID:SCR_008520). Single-stain controls were used for compensation.

### Senescence Associated-β-galactosidase Assay

SKMEL28 cells were treated with 1 µmol/L palbociclib (Sigma-Aldrich, catalog no. PZ0383) for 5 days. Senescence Associated-β-galactosidase (SA-β-Gal) staining was performed as described previously (31). Briefly, cells were fixed in 2% formaldehyde/0.2% glutaraldehyde in PBS (5 minutes) and stained [40 mmol/L Na_2_HPO_4_, 150 mmol/L NaCl, 2 mmol/L MgCl_2_, 5 mmol/L K_3_Fe(CN)_6_, 5 mmol/L K_4_Fe(CN)_6_, and 1 mg/mL X-gal] overnight at 37°C in a non-CO_2_ incubator. Images were acquired at room temperature using an inverted microscope (Nikon Eclipse Ts2) with a 20X/0.40 objective (Nikon LWD) equipped with a camera (Nikon DS-Fi3). Each sample was assessed in triplicate and at least 100 cells per well were counted (>300 cells per experiment).

### qRT-PCR

RNA was collected in TRIzol and isolated as described previously in the “RNA isolation, Sequencing, and Analysis” section. RNA was then retrotranscribed with High-Capacity cDNA Reverse Transcription Kit (Applied Biosystems, catalog no. 4368814) and 20 ng of cDNA amplified using the CFX Connect Real-time PCR system (Bio-Rad) and the PowerUp SYBR Green Master Mix (Applied Biosystems, catalog no. A25742) following manufacturer's instructions. Primers were designed using the Integrated DNA Technologies web tool (Supplementary Table S2). Conditions for amplification were: 5 minutes at 95°C, 40 cycles of 10 seconds at 95°C, and 7 seconds at 62°C. The assay ended with a melting curve program: 15 seconds at 95°C, 1 minute at 70°C, then ramping to 95°C while continuously monitoring fluorescence. Each sample was assessed in triplicate. Relative quantification was determined to multiple reference genes (human: *PSMC4*, and *B2M* and mouse: *Rplp0* and *Gusb*) to ensure reproducibility using the delta-delta CT method.

### Immunofluorescence

An equal number of cells were seeded on coverslips and fixed with 4% paraformaldehyde. Cells were washed out three times with PBS and permeabilized with 0.2% Triton X-100 in PBS for 5 minutes. Cells were blocked for 5 minutes with 3% BSA/PBS followed by incubation with 1/1,000 dilution of anti-phospho-Histone H2A.X (EMD Millipore, catalog no. 05-636) in 3% BSA/PBS for 1 hour at room temperature. Cells were washed three times with 1% Triton X-100 and incubated with cy3 conjugated secondary antibody (Jackson Immunoresearch, catalog no. 712-165-150) diluted 1/1,000 in 3% BSA/PBS and incubated 1 hour at room temperature and in dark. Cells were then incubated with 0.15 µg/mL DAPI for 1 minute, washed three times with PBS, mounted with fluorescence mounting medium [9 mL of glycerol (Thermo Fisher Scientific, catalog no. BP229-1), 1 mL of 1 × PBS, and 10 mg of p-phenylenediamine (EMD Chemicals, catalog no. PX0730); pH was adjusted to 8.0–9.0 using carbonate bicarbonate buffer (0.2 mol/L anhydrous sodium carbonate, 0.2 mol/L sodium bicarbonate)], and sealed. At least 200 cells per coverslip were counted. Cells were considered positive when they contained >10 γH2AX foci.

### *In Vivo* Mouse Experiment

Six to 8 weeks old male athymic NU/J mice were purchased from Jackson Laboratories (RRID:IMSR_JAX:002019). All mice were maintained in a HEPA-filtered ventilated rack system at the Animal Facility of the Assembly Building of The Hillman Cancer Center at the University of Pittsburgh School of Medicine (Pittsburgh, PA). Mice were housed up to 5 mice per cage and in a 12-hour light/dark cycle. All experiments with animals were performed in accordance with institutional guidelines approved by the Institutional Animal Care and Use Committee (IACUC) at the University of Pittsburgh School of Medicine (Pittsburgh, PA). Ten million SKMEL28 shGFP control or shp16 melanoma cells were subcutaneously injected in the right flank of NU/J mice. Mice were monitored daily to identify palpable tumors, after which mice were randomly assigned to vehicle or methotrexate. Mice were treated daily by intraperitoneal injection of vehicle or 10 mg/kg methotrexate (Cayman Chemical Company, catalog no. 13960) diluted in 200 µL PBS. Both mice body weight and tumor [Length (*L*) and width (*W*), where *L* > *W*] were measured three times a week. Tumor volume was calculated as ½ (*L* × *W*^2^). Animals were euthanized once tumors reached 1,000 mm^3^. Tumor volumes were log-transformed to fit a linear model for tumor growth with fixed effects for mouse type (shp16 vs. shcontrol) and for treatment (vehicle vs. methotrexate). Repeated measures on each subject over time were also specified in the model. Growth rates with SEs were calculated using R CRAN.

### Blood Cell Analysis

Fresh whole blood was collected from euthanized mice via the inferior vena cava using a 20-gauge needle (Becton Dickinson, catalog no. 305175) into microvette K2-EDTA–coated tubes (Sarstedt, catalog no. 16.444.100). Total circulating white blood cells were analyzed using an automated ABAXIS VetScan HM5C Hematology analyzer automated blood counter (Allied Analytic, catalog no. 790-0000).

### Analysis of The Cancer Genome Atlas Patient Data

Data were extracted from The Cancer Genome Atlas (TCGA) Skin Cutaneous Melanoma PanCancer Atlas (367 metastatic melanomas) using cBioportal survival analysis (July 2023). Overall survival probability and HRs were calculated using the log-rank statistical test.

### Quantification and Statistical Analysis

GraphPad Prism (version 9.0; RRID:SCR_002798) and RStudio (Version 2023.06.1+524; RRID:SCR_000432) were used to perform statistical analysis. Point estimates with SDs or SEs were reported, as indicated, and the appropriate statistical test was performed using all observed experimental data. All statistical tests performed were two sided and *P* values <0.05 were considered statistically significant.

### Data Availability Statement

The RNA-seq and Poly-seq data generated in this study are publicly available in GEO at GSE243717 and GSE243717. The CRISPR screen data generated in this study are available within the article and its Supplementary Data files.

## Results

### CRISPR Dropout Screens Identify Multiple Nucleotide Metabolism Vulnerabilities in p16/*CDKN2A*^low^ Cells

**FIGURE 1:**
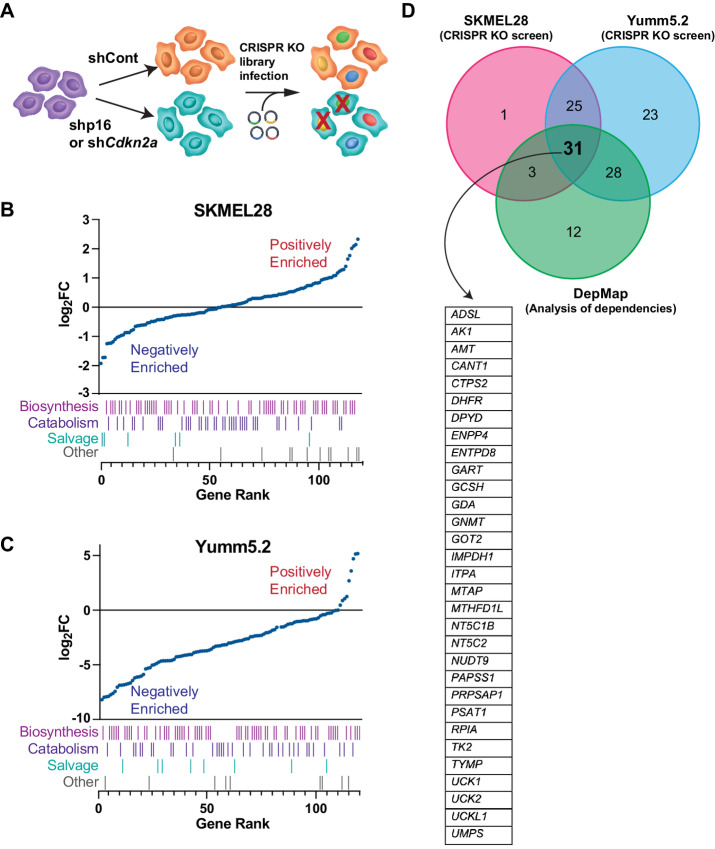
Multiple CRISPR KO screens identify nucleotide metabolism genes that are selectively depleted in p16/*CDKN2A*^low^ cells. **A,** Schematic of our CRISPR screens. p16/*Cdkn2a* wildtype cells were infected with lentiviruses expressing shGFP control (shCont), shp16 (human), or sh*Cdkn2a* (mouse). Human and mouse isogenic cell pairs were infected with nucleotide-focused or whole metabolism-focused CRISPR gRNA libraries, respectively, at an MOI of <0.3. After 14 days in culture, gDNA was harvested and sequenced. Analysis of genes included in the “nucleotide metabolism signature” (Supplementary Table S1) identified multiple genes that are negatively enriched in shp16/sh*Cdkn2a* vs. shCont in human SKMEL28 (**B**) and mouse Yumm5.2 (**C**) melanoma cells. Raw data can be found in Supplementary Tables S3 and S4. **D,** Comparison of datasets and list of 31 common genes negatively enriched (log_2_ fold change <0) in the indicated analyses.

We previously published that knockdown of p16 increases nucleotide synthesis in part via translation of the PPP enzyme RPIA (12). However, there are currently no available RPIA inhibitors; thus, we set out to discover additional nucleotide metabolic enzymes that are vulnerabilities of p16/*CDKN2A*^low^ cells in an attempt to identify pharmacologically targetable pathways. We designed a nucleotide-focused sgRNA library targeting 128 genes directly involved in nucleotide biosynthesis, salvage, and catabolism (referred to as the “nucleotide metabolism signature”; Supplementary Table S1), and performed a dropout screen in SKMEL28 human melanoma cells with wildtype p16 and p16 knockdown (Fig. 1A; Supplementary Fig. S1A). We used a previously validated shp16 hairpin that can be rescued by p16 overexpression, suggesting a lack of off-target effects (12). Notably, this hairpin only targets p16 and not p14 (hairpin information in Materials and Methods). As expected, these cells proliferate faster than isogenic controls (Supplementary Fig. S1B). Using MAGeCK analysis (ref. 25; see Materials and Methods for more details about the screens), we observed negative enrichment for many genes involved in nucleotide homeostasis (Fig. 1B; Supplementary Table S3), including our previously published target *RPIA* (ref. 12; Supplementary Fig. S1C), demonstrating the validity of our screen. We next performed a secondary dropout screen in the Yumm5.2 mouse melanoma isogenic pair with *Cdkn2a* knockdown (Supplementary Fig. S1D), using a cell metabolism-focused library (32). Analyzing genes in the nucleotide metabolism signature defined above, we found that almost all nucleotide metabolism genes were negatively enriched (Fig. 1C; Supplementary Table S4), suggesting a conserved phenomenon between human and mouse cells. Data taken from DepMap using cutaneous melanoma cell lines also indicated increased dependency on several nucleotide metabolism genes based on *CDKN2A* expression (Supplementary Fig. S1E). Using multiple datasets, together these screens identified 31 common genes in both nucleotide catabolism and biosynthesis pathways, including the PPP and one carbon metabolism (Fig. 1D; Supplementary Table S5). These data suggest that multiple nucleotide metabolism pathways are a vulnerability in p16/*CDKN2A*^low^ cells.

### p16/*CDKN2A* Expression Negatively Correlates with Nucleotide Pathway Activity

**FIGURE 2:**
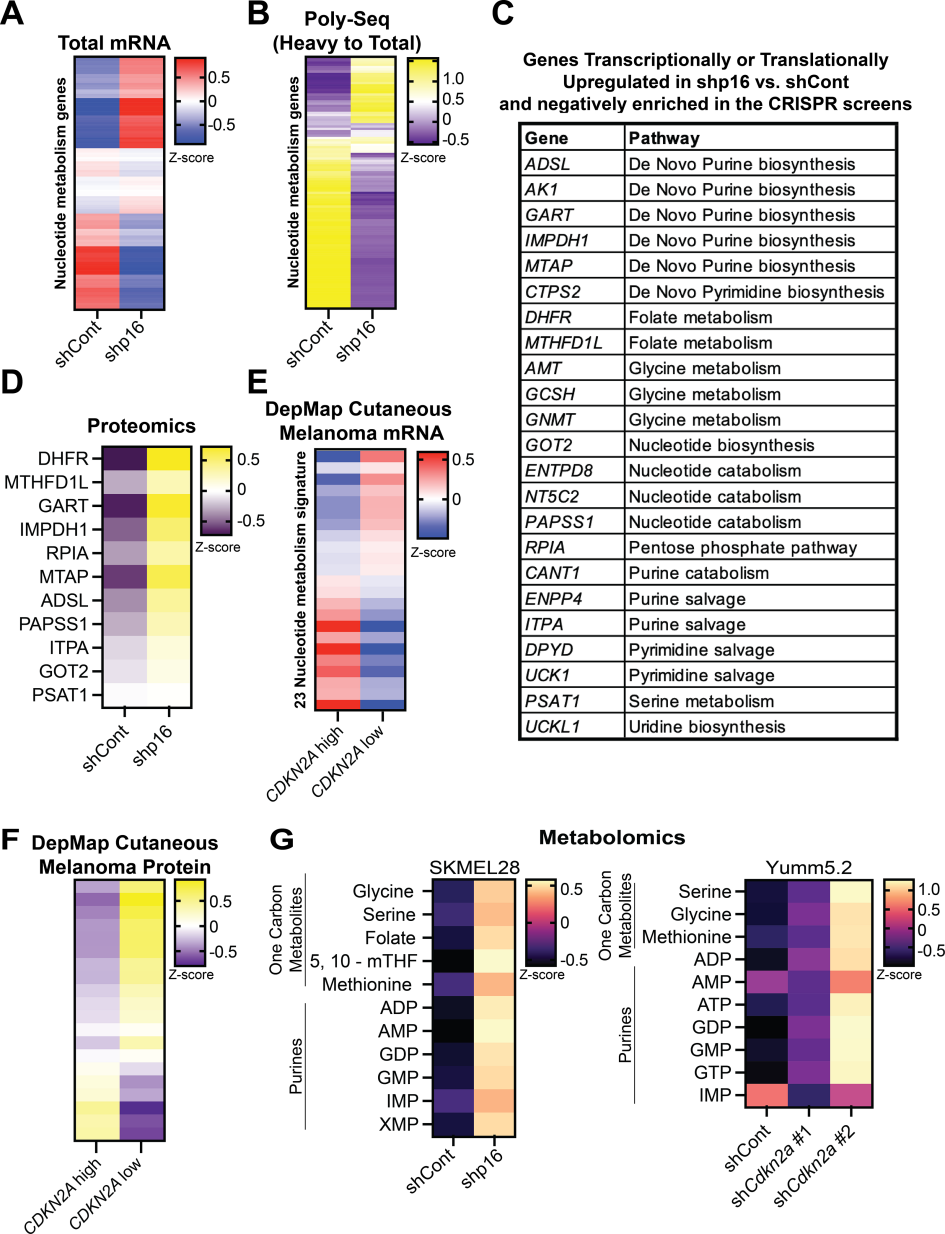
p16/*CDKN2A* negatively correlates with multiple nucleotide metabolism genes, proteins, and metabolites. **A–D,** SKMEL28 human melanoma cells were infected with lentivirus expressing a shRNA targeting p16 (shp16). shGFP was used as a control (shCont). **A,** Expression of the 128 nucleotide metabolism gene signature from RNA-seq. Raw data can be found in Supplementary Table S6. **B,** Polysome fractionation was performed and both the heavy fraction (>2 ribosomes) and total mRNA were sequenced. The ratio of heavy to total was used to assess transcripts with increased translation. Raw data can be found in Supplementary Table S7. **C,** Genes that are transcriptionally or translationally upregulated in shp16 SKMEL28 cells. **D,** Expression of the indicated proteins by proteomics. **E** and **F,** DepMap data of cutaneous melanoma cell lines. **E,** mRNA expression of 23 genes identified in the CRISPR screens. **F,** Protein expression of genes identified in the CRISPR screens. Note only 20 proteins were found in the DepMap data. **G,** Steady-state metabolite profile of one carbon metabolites and purines.

As we observed negative enrichment in both nucleotide synthesis and catabolism genes (Fig. 1), we aimed to further refine potential targetable hits. Toward this goal, we cross-compared our nucleotide metabolism signature with RNA-seq data and found that multiple genes were both depleted in the CRISPR screen and transcriptionally upregulated in shp16 cells (Fig. 2A; Supplementary Table S6). We previously demonstrated that the PPP enzyme RPIA is translationally regulated (12). To investigate whether other nucleotide metabolic proteins also undergo translational regulation, we performed Poly-seq. This analysis identified additional nucleotide metabolism transcripts that are also both depleted in the CRISPR screen and translationally upregulated in shp16 cells (Fig. 2B; Supplementary Table S7). Of the 31 common genes, 23 (∼75%) are either transcriptionally or translationally upregulated in shp16 cells and many correspond to either *de novo* purine synthesis or one carbon metabolism (Fig. 2C). Several of these were validated to be increased in shp16 cells at the protein level (Fig. 2D). Using data from the DepMap, we found many of these 23 genes and proteins correlate with *CDKN2A* mRNA in cutaneous melanoma cell lines (Fig. 2E and F). Consistently, one carbon metabolites and purines were significantly increased in cells upon knockdown of p16/*Ckdn2a* (Fig. 2G). Together, these data demonstrate that *de novo* purine synthesis and one carbon metabolism pathways are upregulated at multiple steps in p16^low^ cells.

### Inhibition of *De Novo* Purine Synthesis and One Carbon Metabolism Decreases Proliferation in p16/*CDKN2A*^low^ Cells

**FIGURE 3:**
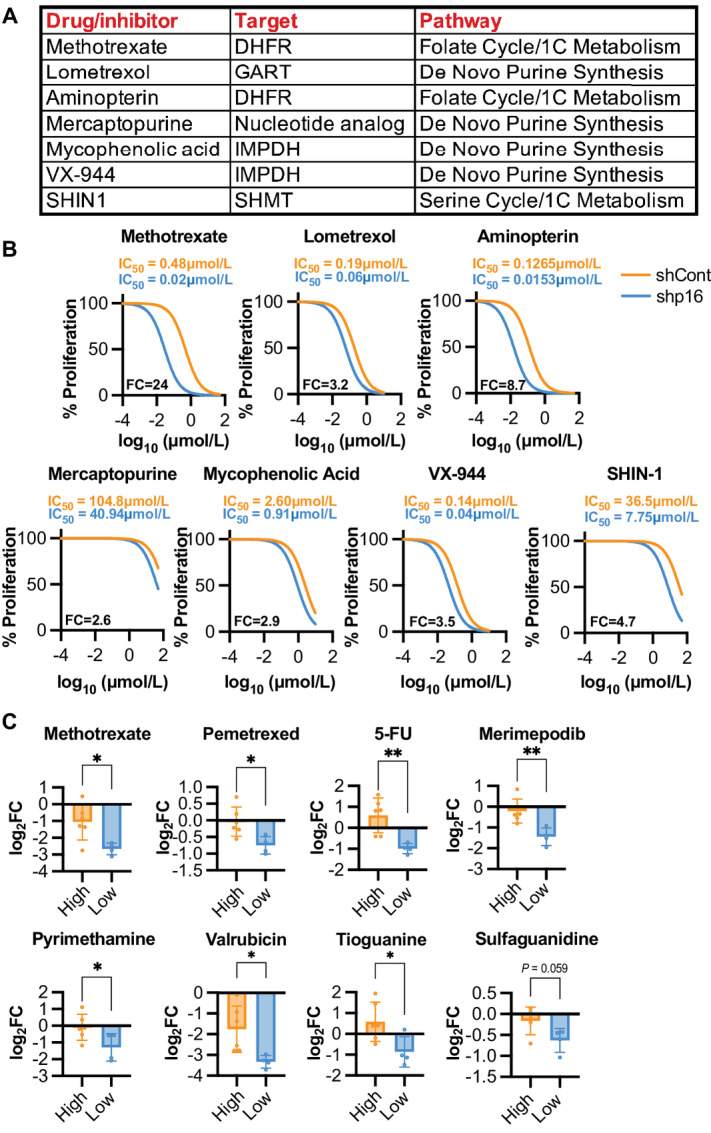
p16/*CDKN2A*^low^ cells are more sensitive to inhibitors of nucleotide metabolism. **A,** Table of inhibitors used in *in vitro* cell line studies. 1C metabolism = one carbon metabolism. **B,** SKMEL28 human melanoma cells were infected with lentivirus expressing a shRNA targeting p16 (shp16). shGFP was used as a control (shCont). Cells were treated with the indicated inhibitors and proliferation was assessed by crystal violet staining. IC_50_ and fold change (shp16 vs. shCont, FC) are indicated. Data from one of 2–3 independent experimental replicates are shown (*n* = 6). **C,** Increased drug sensitivity from DepMap data of cutaneous melanoma cell lines with high or low *CDKN2A* expression. Data are mean ± SD. *t* test. *, *P* < 0.05; **, *P* < 0.01.

Our CRISPR dropout screens reveal that p16/*Cdkn2a* knockdown cells exhibit a vulnerability in multiple nucleotide metabolism pathways (Fig. 1). Further analysis indicates a significant upregulation of *de novo* purine synthesis and one carbon metabolism genes, enzymes, and metabolites in p16/*CDKN2A*^low^ cells (Fig. 2). As our goal is to identify pharmacologic approach to treat these cancer cells, we next used a variety of inhibitors targeting enzymes within these pathways, including several inhibitors of CRISPR screen dropout hits: *DHFR*, *IMPDH*, and *GART* (Fig. 3A), although it is important to note that these inhibitors may have additional targets. SKMEL28 cells with p16 knockdown display decreased proliferation upon inhibition of *de novo* purine synthesis and one carbon metabolism pathways *in vitro* (Fig. 3B). Similar results were observed using cell lines from DepMap and in mouse cells with knockdown or KO of *Cdkn2a* (Fig. 3C; Supplementary Fig. S2A–S2C). Pyrimidine synthesis inhibitors did not have similar effects on p16/*Cdkn2a*^low^ cells (Supplementary Fig. S2D and S2E). For further analysis, we selected the antifolate methotrexate as a lead compound due to its large effect on p16^low^ cells (Fig. 3B). We found that methotrexate increased the percentage of cells in S-phase to a greater extent in both SKMEL28 shp16 and Yumm5.2 sh*Cdkn2a* cells than in p16/*Cdkn2a* wildtype controls (Supplementary Fig. S2F and S2G). Interestingly, knockdown of RB1, which increased proliferation to a similar extent as p16 knockdown (Supplementary Fig. S2H and S2I), did not sensitize cells to antifolates to the same extent as knockdown of p16 (Supplementary Fig. S2J). This suggests a mechanism beyond simply increased proliferation. Finally, while prior reports have demonstrated that deletions of the *MTAP* gene, located in the same chromosomal locus as *CDKN2A* (9p21) and often deleted together (33, 34), increase sensitivity to *de novo* purine inhibitors and antifolates (35–39), we did not observe decreased *MTAP* expression in our p16/*Cdkn2a* knockdown cells (Supplementary Fig. S2K). Altogether, these data validate our CRISPR screen through an orthogonal pharmacologic approach and highlight the essential role of both *de novo* purine synthesis and one carbon metabolism to the proliferation of p16/*CDKN2A*^low^ cells.

### Antifolates Induce Apoptosis in p16/*CDKN2A* Knockdown Cells

**FIGURE 4:**
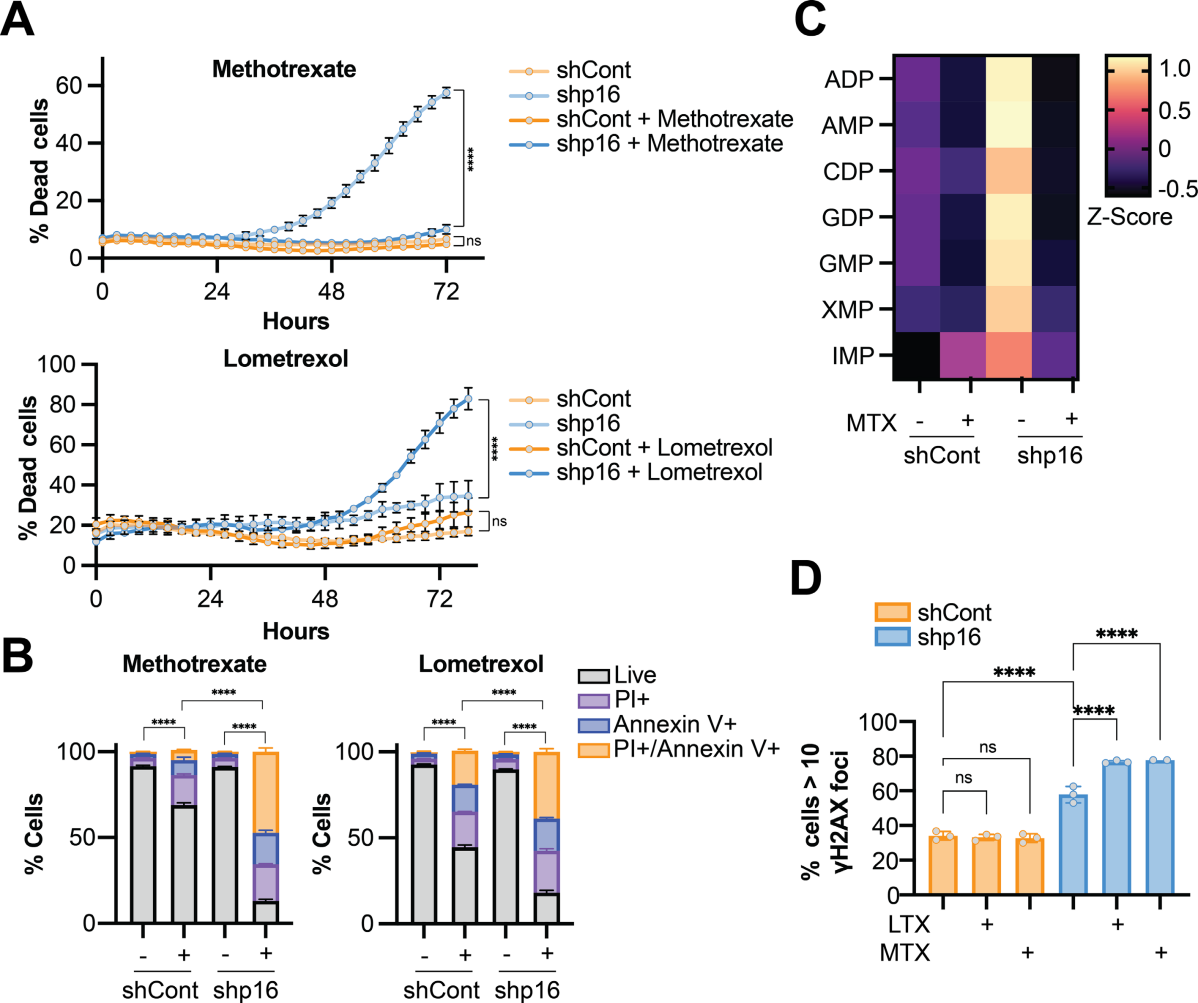
Multiple antifolates induce apoptosis in p16 knockdown cells. **A–D,** SKMEL28 human melanoma cells were infected with lentivirus expressing a shRNA targeting p16 (shp16). shGFP was used as a control (shCont). **A,** Cells were treated with the indicated inhibitors (MTX – 0.17 µmol/L; LTX – 0.12 µmol/L) and cytotoxicity was assessed using IncuCyte Cytotox Green reagent. Data from one of three independent experimental replicates are shown (*n* = 6). Data are mean ± SD. One-way ANOVA at endpoint. ****, *P* < 0.0001. **B,** Cells were treated with the indicated inhibitors (MTX – 0.17 µmol/L; LTX – 0.12 µmol/L) for 72 hours, and apoptosis was assessed using Annexin V/PI (propidium iodide) staining by flow cytometry. Data from one of three independent experimental replicates are shown (*n* = 6). One-way ANOVA of live cells. ****, *P* < 0.0001. **C**, Purine metabolite abundance by mass spectrometry. Cells were treated with methotrexate (MTX; 0.17 µmol/L, 72 hours). Data represent one independent experimental replicate (*n* = 8). Controls are the same data as shown in Fig. 2G. **D,** Cells were treated with lometrexol (LTX; 0.17 µmol/L) or methotrexate (MTX; 0.17 µmol/L) for 72 hours, and immunofluorescence analysis for γH2AX foci was performed. Data from one of two independent experimental replicates are shown (*n* = 3). Data are mean ± SD. One-way ANOVA. ****, *P* < 0.0001; ns = not significant.

Next, we aimed to explore the mechanism of decreased proliferation in p16/*CDKN2A*^low^ cells treated with antifolates. Knockdown of p16/*Cdkn2a* increased cytotoxicity upon antifolate treatment (Fig. 4A; Supplementary Fig. S3A), accompanied by increased apoptosis (Fig. 4B; Supplementary Fig. S3B), although the differences in Yumm5.2 cells were less robust than in SKMEL28. Interestingly, we found that methotrexate depleted purines to a much larger extent in shp16 cells compared with controls (Fig. 4C). We did not observe increased senescence in these cells upon antifolate treatment (Supplementary Fig. S3C). This is in contrast to the CDK4/6 inhibitor palbociclib, which induced robust SA-β-Gal staining in shp16 cells (Supplementary Fig. S3D), although the senescence-associated secretory phenotype (SASP) was blunted (Supplementary Fig. S3E), consistent with our prior report that loss of p16 abrogates the SASP (40). Furthermore, shp16 cells did not exhibit significant proliferation after methotrexate or lometrexol washout; however, they did show increased proliferation upon palbociclib washout (Supplementary Fig. S3F). These data suggest that inhibition of *de novo* purine synthesis via the folate cycle induces apoptosis and not a cytostatic effect. In addition, while CDK4/6 inhibitors are a rational therapeutic option for p16/*CDKN2A*^low^ tumors, our findings suggest that they may not have long-term effects. Many studies have reported a role for the SASP in the antitumor response to CDK4/6 inhibitors (41); therefore, our data have multiple implications for the clinical use of these inhibitors in patients harboring p16/*CDKN2A*^low^ tumors.

To begin to interrogate a mechanism of increased sensitivity of p16^low^ cells to antifolates, we explored two possibilities: folate transporter expression and DNA damage. Interestingly, we observed increased expression of folate transporters in p16^low^ cells (Supplementary Fig. S3G), although the specific transporter was different in human versus mouse cells. As these proteins also transport antifolates (42), it is possible that this is one of the mechanisms of increased sensitivity. Finally, in our recent report, we found that knockdown of p16/*Cdkn2a* increases DNA damage foci (11). Methotrexate further exacerbated DNA damage in cells with knockdown of p16/*Cdkn2a* (Fig. 4D; Supplementary Fig. S3H). These data demonstrate that p16/*CDKN2A*^low^ cells have enhanced DNA damage upon antifolate treatment and provide insights into the potential mechanisms driving the increase in apoptosis in these cells.

### Knockdown of p16 *In Vivo* Decreases Tumor Burden in Response to the Antifolate Methotrexate

**FIGURE 5:**
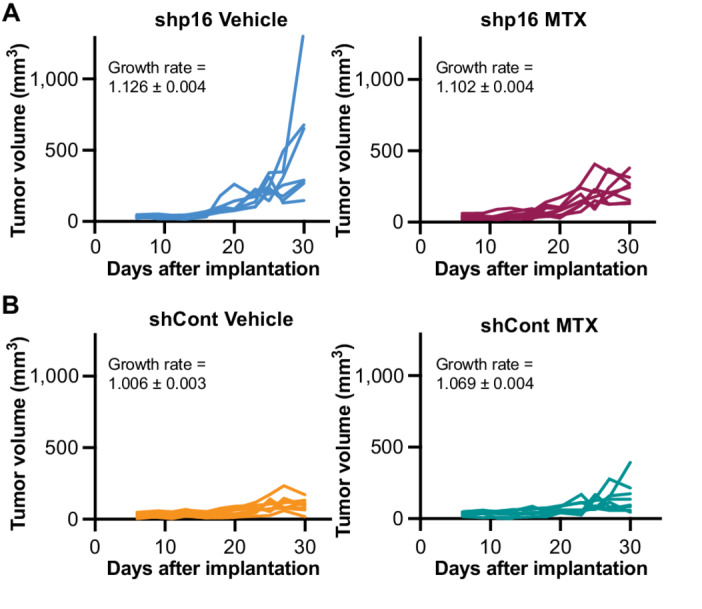
shp16 tumors are more sensitive to the antifolate methotrexate. **A** and **B,** SKMEL28 human melanoma cells were infected with lentivirus expressing a shRNA targeting GFP (shCont) or p16 (shp16). A total of 10^7^ cells were subcutaneously implanted into athymic nude mice. Mice were treated with vehicle controls or methotrexate (MTX). Individual tumor growth curves in shp16 tumors (**A**) and shCont tumors (**B**). Shown are growth rates ± SE. Linear mixed-model group comparisons: shp16 versus shCont *P* < 0.001; shCont: MTX versus vehicle *P* = 0.734; shp16: MTX versus vehicle *P* = 0.031; (shp16: MTX vs. vehicle) versus (Control: vehicle vs. MTX) *P* = 0.002.

We observed decreased proliferation, increased metabolites from one carbon metabolism, and increased in death of shp16 cells treated with the antifolate methotrexate (Figs. 2 and 4). Thus, we determined the effects of methotrexate in subcutaneous SKMEL28 tumors with and without p16 knockdown. We observed a significant difference in tumor growth rate between shp16 tumors treated with methotrexate versus vehicle controls (*P* = 0.031) at day 30 when the first vehicle-treated mice reached IACUC endpoints (1,000 mm^3^). We also observed a trend toward increased survival in mice bearing shp16 tumors treated with methotrexate (Fig. 5A; Supplementary Fig. S4A). Control tumors did not show appreciable differences in growth between treatment and vehicle control (*P* = 0.734), and mice bearing control tumors did not have a significant difference in survival between vehicle and methotrexate treatment groups at the time the shp16 tumors all reached endpoint (Fig. 5B; Supplementary Fig. S4B). Notably, control tumors show a slower growth compared with shp16 tumors, which may impair methotrexate efficacy. Interestingly, comparison between control tumors and shp16 methotrexate-treated tumors was highly significant both at day 30 (*P* = 0.002) and at the timepoint when all shp16 mice had reach endpoint (day 55, *P* < 0.001), although we do note variability in this experiment (Supplementary Fig. S4C and S4D). Methotrexate is known to have side effects, including decreased white blood cell counts (43). We did not observe differences in body weight or blood cell counts in mice bearing shp16 tumors treated with methotrexate at the dose and duration used (Supplementary Fig. S4E and S4F). These data provide evidence that methotrexate may be clinically beneficial for p16/*CDKN2A*^low^ tumors, although additional experiments with fine-tuned methotrexate regimens and additional models are needed.

### *De Novo* Purine Synthesis and One Carbon Metabolism Genes Correspond to Worse Melanoma Patient Survival

**FIGURE 6:**
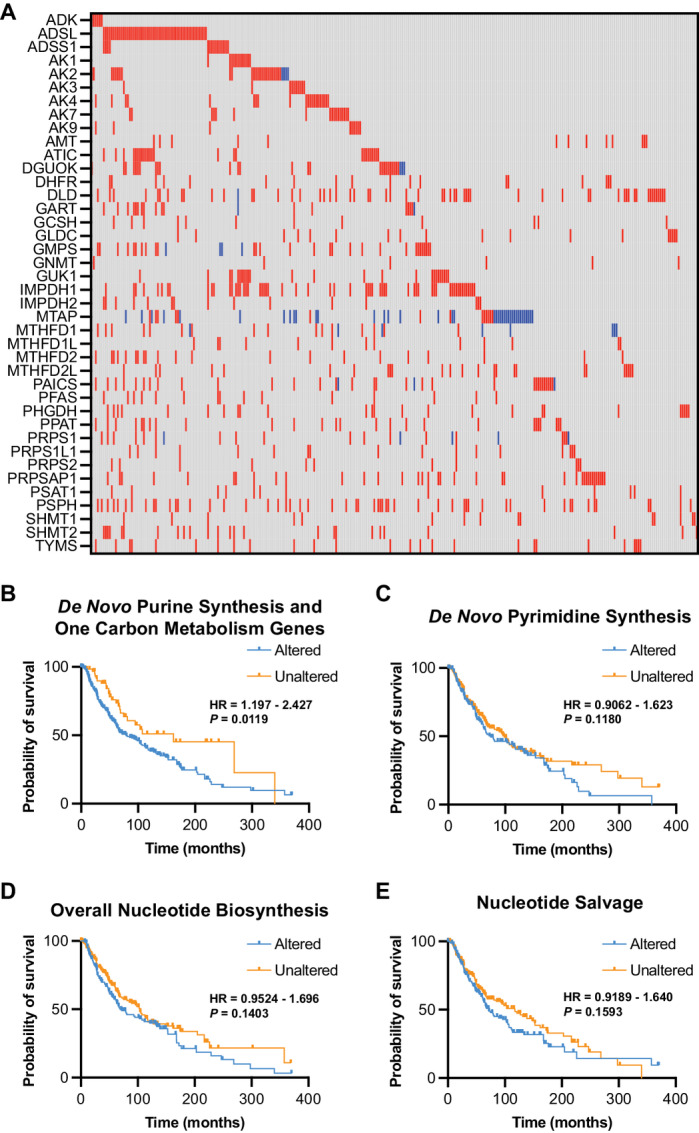
*De novo* purine synthesis and one carbon metabolism genes are associated with worse overall survival in metastatic melanomas. Data from TCGA Skin Cutaneous Melanoma PanCancer Atlas (367 metastatic melanomas). Raw data can be found in Supplementary Table S8. **A,***De novo* purine synthesis and one carbon metabolism genes in TCGA metastatic melanoma samples. Red indicates increased mRNA expression. Blue indicates decreased mRNA expression. Overall survival probability of patients with alterations in *de novo* purine synthesis and one carbon metabolism genes (**B**), *de novo* pyrimidine genes (**C**), overall nucleotide biosynthesis genes (**D**), and nucleotide salvage genes (**E**). log-rank *P*-value and 95% confidence interval.

Finally, using TCGA data, we found that upregulation of *de novo* purine synthesis and one carbon metabolism genes occurs in many metastatic melanomas, although *MTAP* is typically downregulated because of deletion of chromosome 9p21 (ref. 44; Fig. 6A; Supplementary Table S8). Moreover, alteration of these genes corresponds with worse overall survival (Fig. 6B), whereas upregulation of genes involved in *de novo* pyrimidine synthesis, overall nucleotide biosynthesis, or nucleotide salvage does not have an association with melanoma patient survival (Fig. 6C–E). Together, these data show that alterations in genes related to *de novo* purine synthesis and one carbon metabolism are specifically associated with melanoma patient survival.

## Discussion

We previously found that p16 loss increases nucleotide synthesis via mTORC1 and the PPP (12). Here we identified multiple other nucleotide metabolic enzymes that are potential therapeutically targetable vulnerabilities of p16/*CDKN2A*^low^ cells. Upon direct comparison of multiple antifolates and antimetabolites in both human and mouse isogenic pairs with knockdown of p16 and sh*Cdkn2a*, we found that p16 status corresponds to therapeutic response to many of these agents. These agents induce cell death, unlike the CDK4/6 inhibitor palbociclib that induces senescence. Given the context-dependent effects of senescence on the microenvironment (detailed below), these data suggest that *de novo* purine synthesis or one carbon metabolism inhibitors may provide therapeutic benefit for the approximately 50% of p16/*CDKN2A*^low^ human tumors.

Previous studies and clinical trials found that most chemotherapies have little clinical benefit in patients with melanoma (45). However, to date, trials have not stratified patients based on p16/*CDKN2A* expression. To our knowledge, there is only one study that shows a potential susceptibility to antimetabolites, including methotrexate in p16/*CDKN2A*-altered astrocytic tumors (46). While alterations of p16/*CDKN2A* are widely reported in melanoma (47, 48), there is a lack of p16/*CDKN2A* assessment in the routine clinical practice unless hereditary melanoma is suspected (49). Notably, recent surveys conducted among dermatologists, pathologists, and general practitioners from different countries including the United States indicate that only 7.3% of clinicians routinely assess p16 status for their diagnoses (50). The reasons for the lack of p16 assessment is not clear but may be due in part to complexities arising from the heterogeneity and non-binary nature of p16 IHC staining (51). Our data suggest that while patients with wildtype p16/*CDKN2A* or high to moderate p16/*CDKN2A* expression would likely not robustly respond to antimetabolite therapies, those with low or mutant p16/*CDKN2A* may benefit. In addition, we acknowledge that methotrexate has not shown clinical efficacy in melanoma, and therefore other factors beyond p16/*CDKN2A* status are likely at play. It is possible that this finding is more relevant in cancers other than melanoma. Indeed, we have previously published that our work on p16 loss is universal to many cancer types (12, 40). Moreover, the *CDKN2A* gene, located on chromosome 9p21 is often deleted along with neighboring genes including *MTAP* (44). Prior work has found that patients with *MTAP* deletions are more responsive to *de novo* purine synthesis inhibitors, including antifolates (35, 36, 52). However, we did not observe a decrease in *MTAP* expression upon p16 knockdown (Supplementary Fig. S2K), suggesting that suppression of p16/*CDKN2A* itself may contribute to increased sensitivity to *de novo* purine and one carbon metabolism inhibitors. Thus, cancers with homozygous deletion of the entire locus containing both genes would presumably be highly sensitive to these inhibitors, although this will need to be tested experimentally in isogenic cells. In addition, we previously demonstrated that p16 loss increases mTORC1 activity (12), and a recent study found that cancers with increased mTORC1, such as those with TSC2 deficiency, are highly sensitive to inhibitors of IMPDH (53). Both studies suggest mTORC1 pathway as potential mechanism to regulate the purine metabolism in p16^low^ cells. Together, these findings underscore the importance of reevaluating whether patient stratification based on p16/*CDKN2A* status should be regularly incorporated into clinical practice for future antimetabolite therapy.

Multiple antifolates increased cell death in cells with p16 or *Cdkn2a* knockdown (Fig. 4; Supplementary Fig. S3), although the precise mechanism remains to be determined. p16 knockdown cells proliferate faster than p16 wildtype cells (Supplementary Fig. S1B). Increased proliferation would increase the demand for dNTPs and likely other nucleotides required for biomass. Indeed, many chemotherapies are more effective in highly proliferative cells, and the status of other cell cycle regulators affect response to antifolates and antimetabolites (54–56). Interestingly, we found that RB1 knockdown also increased proliferation of cells but did not markedly affect sensitivity to antifolates (Supplementary Fig. S2I and S2J). This is similar to our prior work showing that p16 has roles outside of the canonical RB pathway (12). We cannot rule out other effects of these agents, such as redox imbalance due to inhibition of glutathione synthesis or NADPH, changes in methylation due to methionine cycle dysregulation, or energy stress due to decreased ATP. Indeed, one article has linked p16 deficiency to increased mitochondrial reactive oxygen species that is uncoupled from cell cycle regulation (57), again suggesting a need to better understand noncanonical roles of p16 when mechanistically investigating therapies. Another recent article found that imbalanced nucleotides decrease proliferation (58); thus, it is also possible that the antimetabolites used here create a nucleotide imbalance that perturb S-phase. Whether methotrexate killing activity in p16/*CDKN2A*^low^ cells is only due to decreased nucleotide or additional pathways will be determined in future studies. We did identify three intriguing potential mechanisms to explain the increased sensitivity in p16^low^ cells. First, there is a marked increase in one carbon metabolites and purines in shp16 cells compared with controls, and methotrexate depletes purines to a greater extent in these cells (Figs. 2G and 4C). Second, expression of folate transporters is increased in p16/*Cdkn2a*^low^ cells (Supplementary Fig. S3G). Thus, it is possible that antifolates are taken up by these cells to a greater extent. Finally, we found an increase in basal DNA damage foci in p16/*Cdkn2a*^low^ cells (11) that was exacerbated by antifolates (Fig. 4D; Supplementary Fig. S3H). These data are consistent with prior work on DNA damage and sensitivity to antifolates (59, 60). All these data point to the enhanced cell cycle disruption and sensitivity to these agents observed in p16/*Cdkn2a*^low^ cells. Future work will be required to more fully elucidate the mechanisms of sensitivity, which may provide additional evidence into the metabolic reprogramming induced by p16/*CDKN2A* loss. Interestingly, recent reports have demonstrated that media and FBS conditions are critically important for response to methotrexate (61, 62). Moreover, depleting folate may increase sensitivity to antifolates (63, 64). In this regard, it is interest to speculate that depleting folate in the media or diet may more robustly affect p16/*Cdkn2a*^low^ cells either alone or in combination with antifolates. While outside the scope of the present study, future studies will be aimed at understanding how diet, and dietary folate specifically, affects p16^low^ tumors.

Melanoma standard-of-care is now immunotherapy (65–67), although >40% of patients do not achieve a significant response (66–70). Interestingly, recent data demonstrate that a decreased purine to pyrimidine ratio leads to a nucleotide imbalance that elevates the expression of the immunoproteasome and enhances anti-PD1 response (71, 72). Another study found that the antifolate pemetrexed induces immunogenic cell death and augments T-cell function to synergize with anti-PD1 (73). Therefore, these inhibitors may promote an antitumor immune response that could be exploited in combination with immunotherapy. Indeed, multiple trials are ongoing to better understand whether these agents can be used in combination in a variety of solid tumors, including melanoma (ref. 9; clinicaltrials.gov). While these drugs are also potent immunosuppressants, our data suggest that there is a therapeutic window in p16/*CDKN2A*^low^ tumors that may allow for an antitumor response that does not have marked effects on blood cell counts (Supplementary Fig. S4F), although additional experiments are needed.

CDK4/6 inhibitors are currently in clinical trials in combination with immunotherapies in melanoma and other solid tumors (refs. 9, 74–76; clinicaltrials.gov). These inhibitors induce cytostasis, generally associated with a senescence arrest (77). Indeed, we observed increased SA-β-Gal activity in shp16 cells treated with the CDK4/6 inhibitor palbociclib (Supplementary Fig. S3D). However, cells began to proliferate again after washout of palbociclib, suggesting that this is either a pseudosenescent phenotype or that a population of cells that do not senesce are capable of repopulating after discontinuing therapy. Induction of senescence may promote an antitumor immune response via the SASP (78–82), although other studies suggest senescence and the SASP is immunosuppressive (83–85). We previously published that loss of p16/*CDKN2A* expression corresponds to decreased SASP (40), and our data here indicate a blunted SASP induction in p16 knockdown cells treated with palbociclib (Supplementary Fig. S3E). This suggests that while CDK4/6 inhibitors induce a potent cytostatic effect in p16^low^ cells, they may not have the added benefit to potentiate immunotherapy due to suppressed proinflammatory factors. Experiments to determine the extent of synergy between CDK4/6 inhibitors and immunotherapy in the context of p16/*CKDN2A* expression will be helpful toward understanding this combination.

While our study successfully uncovered the correlation between p16/*CDKN2A* status and the therapeutic response to various inhibitors of *de novo* purine synthesis pathway including antifolates, some limitations of this study warrant acknowledgement. First, this study is based on CRISPR screens but use pharmacologic agents for validation, some of which are known to work via polypharmacology*.* Second, this study is limited in cellular models. However, our strategic inclusion of two cell lines from different species (human and mouse) increase the biological significance of our findings through a cross-species validation suggesting a conserved mechanism. Moreover, this observation paves the way to explore antifolates in other cancer cell types with low p16 expression. Finally, although we demonstrate a robust *in vitro* response, our *in vivo* experiment showed a modest and variable response of shp16 tumors to methotrexate. Future studies using additional animal models and fine-tuned therapeutic regimens—such as changing folate in the diet—are necessary to further understand the effect of antimetabolites *in vivo*.

In summary, we identified multiple metabolic vulnerabilities of p16/*CDKN2A*^low^ cancer cells that can be exploited using various antimetabolites or antifolates. While we focused on melanoma cells, these results may have implications for other human tumors where p16/*CDKN2A* expression is lost as we have previously published that metabolic changes due to p16 loss are a universal phenomenon (12). Low expression of p16/*CDKN2A* may open up a therapeutic window for these agents that kill tumor cells, although this will need to be further investigated in humans.

## Supplementary Material

Figure S1 Knockdown of p16 or Cdkn2a in human and mouse melanoma cell lines, respectively, and DepMap dependency score data based on CDKN2A expression. Related to Figure 1.

Figure S2 Knockdown or knockout of Cdkn2a in mouse melanoma cell lines increases sensitivity to multiple anti-folates but not to de novo pyrimidine synthesis; Knockdown of RB1 does not recapitulate the anti-folate response exhibited by shp16 cells. Related to Figure 3.

Figure S3 Anti-folates induce death and increased DNA damage foci in shCdkn2a cells; shp16 cells do not mount a robust senescence-associated secretory phenotype (SASP) in response to Palbociclib; anti-proliferative effects of anti-folates are longer-lived than palbociclib; and folate transporters are upregulated in shp16/shCdk2na cells. Related to Figure 4.

Figure S4 shp16 tumor bearing mice treated with methotrexate have a trend towards a survival advantage; and methotrexate does not affect body weight or blood cell counts. Related to Figure 5.

Table S1 CRISPR KO library containing 128 genes comprising the nucleotide metabolism signature

Table S2 Primers used in this study

Table S3 CRISPR screen results for human SKMEL28 cells

Table S4 CRISPR screen results for mouse Yumm5.2 cells

Table S5 31 common genes identified in screens and publicly available data

Table S6 mRNA expression of nucleotide metabolism genes

Table S7 Poly-Seq data of nucleotide metabolism genes

Table S8 TCGA melanoma patient data
