# Supplementary material for: De Novo Purine Metabolism is a Metabolic Vulnerability of Cancers with Low p16 Expression
Source: Cancer Res Commun. 2024 May 2;4(5):1174–88. doi: 10.1158/2767-9764.CRC-23-0450 (PMC11064835; doi:10.1158/2767-9764.CRC-23-0450)
Supplement: Figure S1 — Knockdown of p16 or Cdkn2a in human and mouse melanoma cell lines, respectively, and DepMap dependency score data based on CDKN2A expression. Related to Figure 1. [file crc-23-0450-s01.pdf]

Supplemental Figure 1

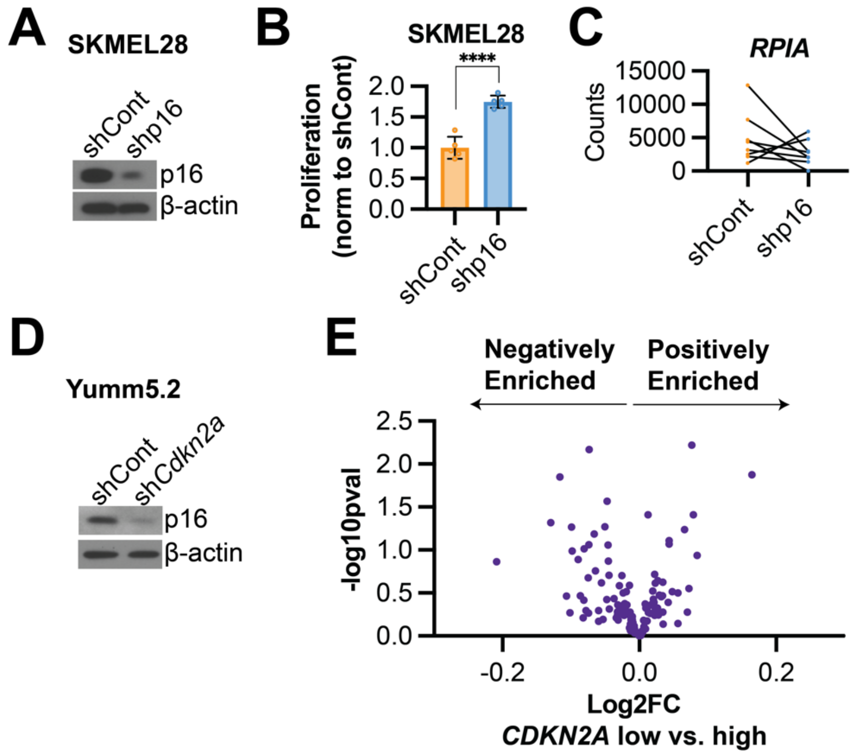

Figure S1. Knockdown of p16 or *Cdkn2a* in human and mouse melanoma cell lines, respectively, and DepMap dependency score data based on *CDKN2A* expression. Related to Figure 1. (A-B) SKMEL28 human melanoma cells were infected with lentivirus expressing a short hairpin RNA (shRNA) targeting p16 (shp16- blue). shGFP was used as a control (shCont- orange). **(A)** Immunoblot analysis of p16. β-actin was used as a loading control. Data from one of 6 independent experimental replicates is shown. **(B)** Proliferation was assessed by crystal violet staining. One of 6 independent experiments is shown (n=6). Data are mean ± SD. T-test. ****p<0.0001 **(C)** Counts for each *RPIA* gRNA in the SKMEL28 CRISPR KO screen (**see Fig. 1B**). **(D)** Yumm5.2 mouse melanoma cells were infected with lentivirus expressing a shRNA targeting *Cdkn2a* (sh*Cdkn2a*). shGFP was used as a control (shCont). Immunoblot analysis of p16. β-actin was used as a loading control. Data from one of 3 independent experimental replicates is shown. **(E)** Volcano plot of dependency data from DepMap of cutaneous melanoma cell lines based on *CDKN2A* expression.
