# Supplementary material for: De Novo Purine Metabolism is a Metabolic Vulnerability of Cancers with Low p16 Expression
Source: Cancer Res Commun. 2024 May 2;4(5):1174–88. doi: 10.1158/2767-9764.CRC-23-0450 (PMC11064835; doi:10.1158/2767-9764.CRC-23-0450)
Supplement: Figure S2 — Knockdown or knockout of Cdkn2a in mouse melanoma cell lines increases sensitivity to multiple anti-folates but not to de novo pyrimidine synthesis; Knockdown of RB1 does not recapitulate the anti-folate response exhibited by shp16 cells. Related to Figure 3. [file crc-23-0450-s02.pdf]

Supplemental Figure 2

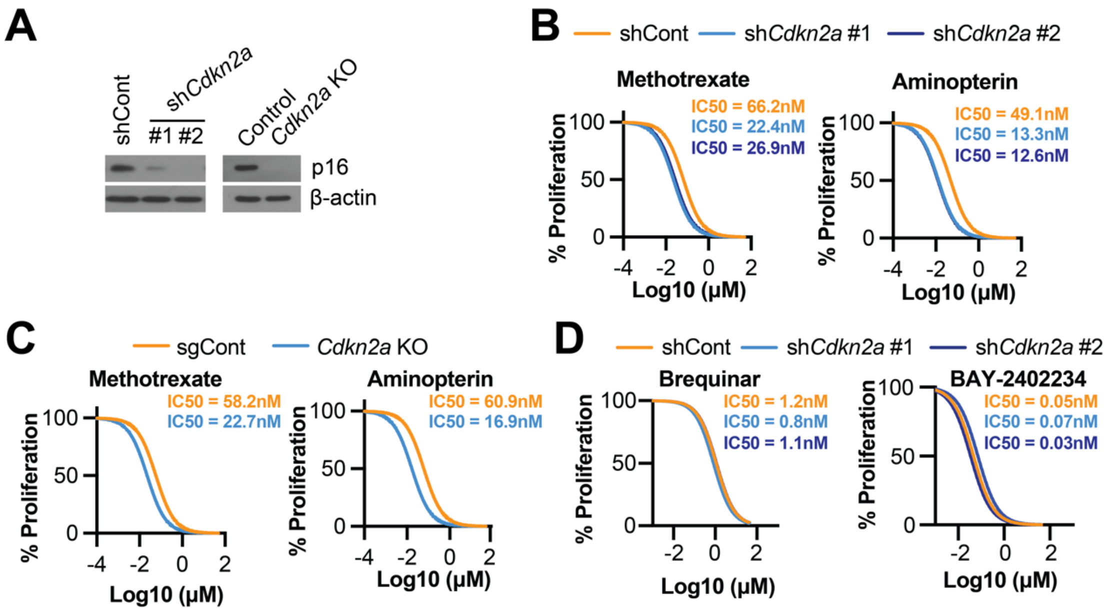

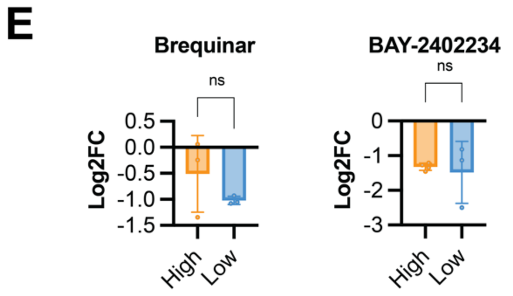

F

SKMEL28 Cell cycle profile

	G0/G1	S	G2/M
shCont DMSO	72.00 (±0.62)	14.43 (±0.99)	10.58 (±1.09)
shCont MTX	59.07 (±0.42)	23.87 (±0.40)	9.11 (±0.28)
shp16 DMSO	62.27 (±0.91)	18.53 (±0.91)	15.87 (±0.85)
shp16 MTX	23.98 (±15.18)	54.57 (±13.90)	8.62 (±5.70)

G

Yumm5.2 Cell cycle profile

	G0/G1	S	G2/M
shCont DMSO	57.13 (±4.48)	22.30 (±4.35)	15.74 (±3.68)
shCont MTX	43.37 (±1.04)	32.60 (±0.85)	10.08 (±0.82)
sh*Cdkn2a*#1 DMSO	60.00 (±5.60)	25.33 (±2.87)	11.10 (±2.61)
sh*Cdkn2a*#1 MTX	31.80 (±1.61)	35.33 (±1.12)	19.43 (±12.93)
shCdkn2a #2 DMSO	54.20 (±0.95)	26.17 (±1.40)	34.10 (±5.50)
shCdkn2a #2 MTX	28.57 (±0.67)	41.37 (±0.15)	13.20 (±0.46)

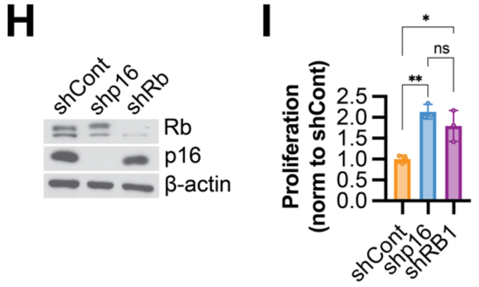

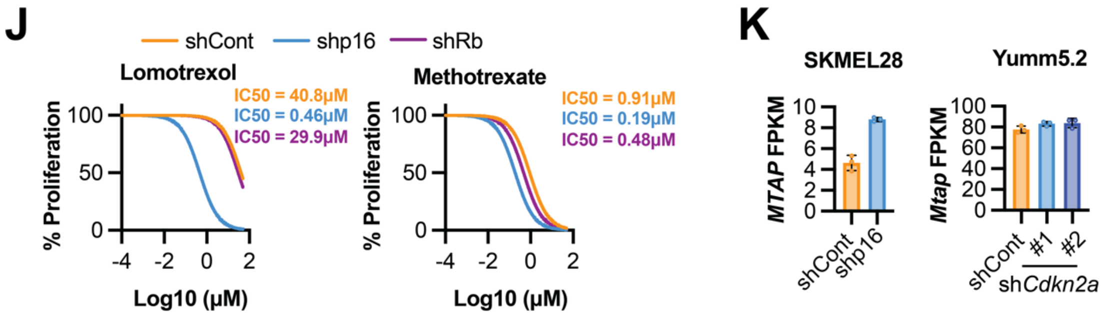

Figure S2. Knockdown or knockout of *Cdkn2a* in mouse melanoma cell lines increases sensitivity to multiple anti-folates but not to *de novo* pyrimidine synthesis; Knockdown of RB1 does not recapitulate the anti-folate response exhibited by shp16 cells. Related to Figure 3. (A-C) Yumm5.2 mouse melanoma cells were infected with lentivirus expressing short hairpin RNAs (shRNA) targeting *Cdkn2a* (sh*Cdkn2a* #1- light blue; sh*Cdkn2a* #2- dark blue) or a gRNA targeting *Cdkn2a* (light blue). shGFP was used as a control for KD experiments (shCont- orange). A gRNA targeting an intergenic region was used as a control for KO experiments. **(A)** Immunoblot analysis of p16. β-actin was used as a loading control. Data from one of 3 independent experimental replicates is shown. **(B-C)** Cells were treated with the indicated inhibitors, and proliferation was assessed by crystal violet staining. Data from one of 2-3 independent experimental replicates is shown (n=3). **(E)** No significant difference in drug sensitivity from DepMap data of cutaneous melanoma cell lines with high or low *CDKN2A* expression. Data are mean ± SD. T-test. ns = not significant. **(F-G)** Cells were treated with methotrexate (SKMEL28 – 0.17μM, Yumm5.2 – 22nM) for 72h, and cell cycle was assessed using propidium iodide staining. Data are mean ± SD. Data from one of 2 independent experimental replicates is shown (n=3). **(H-J)** SKMEL28 human melanoma cells were infected with lentivirus expressing a short hairpin RNA (shRNA) targeting p16 (shp16- blue) or RB1 (shRB1- purple). shGFP was used as a control (shCont- orange). **(H)** Immunoblot analysis of p16 and RB1. β-actin was used as a loading control. Data from one of 3 independent experimental replicates is shown. **(I)** Proliferation was assessed by crystal violet staining. One of 3 independent experiments is shown (n=4). Data are mean ± SD. One-way ANOVA. **(J)** Cells were treated with the indicated inhibitors, and proliferation was assessed by crystal violet staining. Data from one of 3 independent experimental replicates is shown (n=3). **(K)** *MTAP* Fragments Per Kilobase of transcript per Million mapped reads (FPKM) in SKMEL28 human melanoma cells with p16 knockdown (shp16) and Yumm5.2 mouse melanoma cells with *Cdkn2a* knockdown (sh*Cdkn2a* #1 and #2). Data are mean ± SD. *p<0.05; **p<0.01; ns = not significant.
