# Supplementary material for: De Novo Purine Metabolism is a Metabolic Vulnerability of Cancers with Low p16 Expression
Source: Cancer Res Commun. 2024 May 2;4(5):1174–88. doi: 10.1158/2767-9764.CRC-23-0450 (PMC11064835; doi:10.1158/2767-9764.CRC-23-0450)
Supplement: Figure S3 — Anti-folates induce death and increased DNA damage foci in shCdkn2a cells; shp16 cells do not mount a robust senescence-associated secretory phenotype (SASP) in response to Palbociclib; anti-proliferative effects of anti-folates are longer-lived than palbociclib; and folate transporters are upregulated in shp16/shCdk2na cells. Related to Figure 4. [file crc-23-0450-s03.pdf]

Supplemental Figure 3

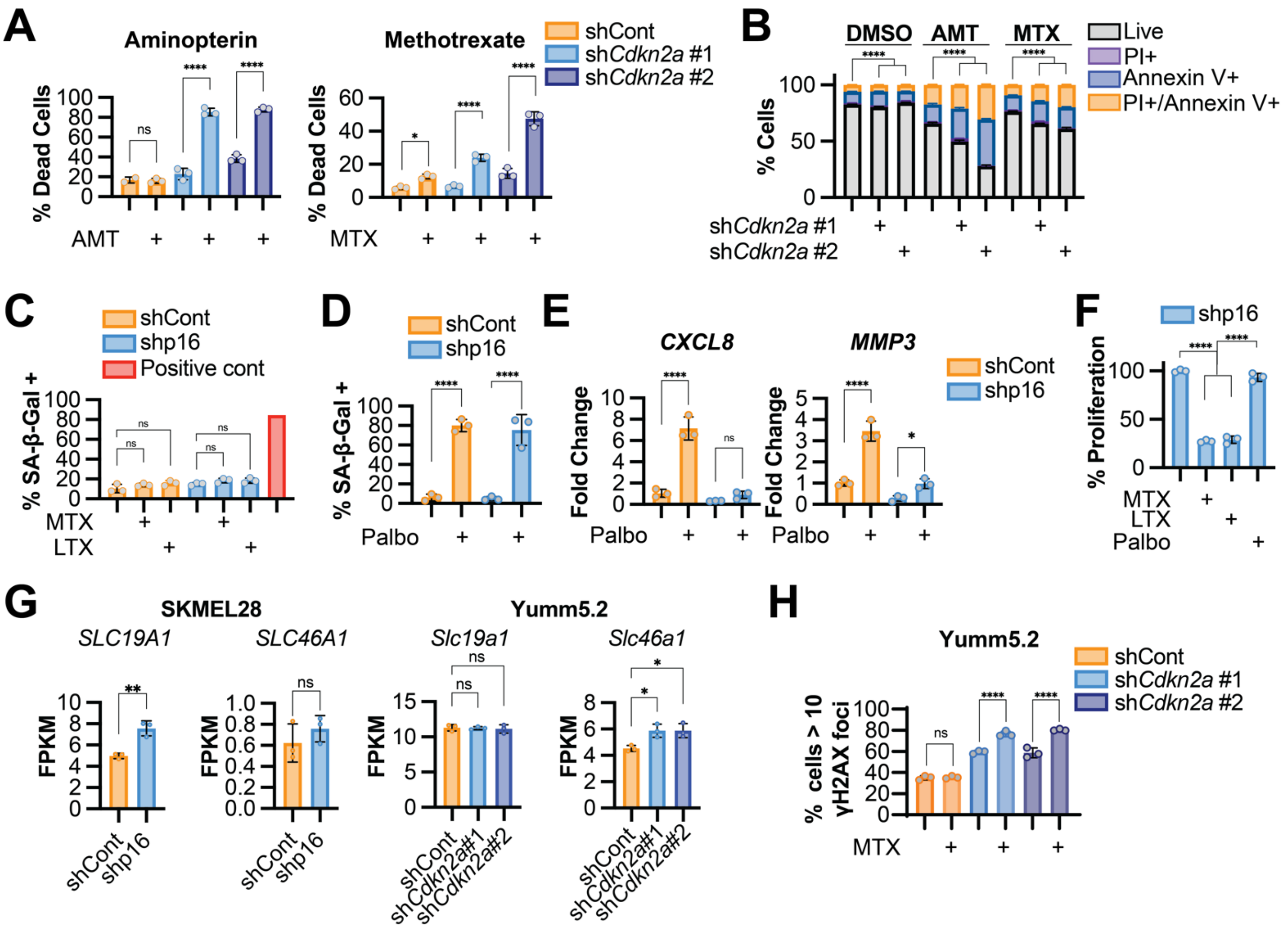

Figure S3. Anti-folates induce death and increased DNA damage foci in sh*Cdkn2a* cells; shp16 cells do not mount a robust senescence-associated secretory phenotype (SASP) in response to Palbociclib; anti-proliferative effects of anti-folates are longer-lived than palbociclib; and folate transporters are upregulated in shp16/sh*Cdk2na* cells. Related to Figure 4. (A-B, G, H) Yumm5.2 mouse melanoma cells were infected with lentivirus expressing short hairpin RNAs (shRNA) targeting *Cdkn2a* (sh*Cdkn2a* #1- light blue; sh*Cdkn2a* #2- dark blue). shGFP was used as a control for KD experiments (shCont- orange). **(A)** Cells were treated with the indicated inhibitors (AMT – 10nM, MTX – 25nM) for 72h, and cytotoxicity was assessed using IncuCyte Cytotox reagent. Data from one of 2 independent experimental replicates is shown (n=3). **(B)** Cells were treated with the indicated inhibitors (AMT – 13nM, MTX – 22nM) for 72h, and apoptosis was assessed using Annexin V/PI staining by flow cytometry. Data from one of 2 independent experimental replicates is shown (n=3). Statistical analysis of live cells is shown. **(C-G)** SKMEL28 human melanoma cells were infected with lentivirus expressing a short hairpin RNA (shRNA) targeting p16 (shp16- blue). shGFP was used as a control (shCont- orange). **(C)** Cells were treated with the indicated inhibitors (MTX – 0.17μM; LTX – 0.12μM) for 72h, and senescence-associated beta-galactosidase (SA-β-Gal) activity was assessed. Cisplatin was used as a positive control (red). Data from one of 3 independent experimental replicates shown (n=3). **(D-E)** Cells were treated with 1μM palbociclib for 7 days, and SA-β-Gal activity **(D)** and SASP gene expression **(E)** was quantified. Data from one of 3 independent experimental replicates is

shown (n=3). **(F)** shp16 cells were treated with the indicated drugs (MTX – 0.17μM; LTX – 0.12μM) for 4 days, after which the drugs were washed out. After an additional 5 days in culture, proliferation was assessed by crystal violet staining. Data from one of 2 independent experimental replicates is shown (n=3). **(G)** Folate transporter mRNA expression in the indicated cells. **(H)** Yumm5.2 cells were treated with methotrexate (22nM) for 72h, and immunofluorescence analysis for γH2AX foci was performed. Data from one of 2 independent experimental replicates is shown (n=3). Data are mean ± SD. One-way ANONA. *$p<0.05$; ***$p<0.001$; ****$p<0.0001$; ns = not significant. AMT: aminopterin; MTX: methotrexate; LTX: lomotrexol; palbo: palbociclib.
