# Supplementary material for: De Novo Purine Metabolism is a Metabolic Vulnerability of Cancers with Low p16 Expression
Source: Cancer Res Commun. 2024 May 2;4(5):1174–88. doi: 10.1158/2767-9764.CRC-23-0450 (PMC11064835; doi:10.1158/2767-9764.CRC-23-0450)
Supplement: Figure S4 — shp16 tumor bearing mice treated with methotrexate have a trend towards a survival advantage; and methotrexate does not affect body weight or blood cell counts. Related to Figure 5. [file crc-23-0450-s04.pdf]

Supplemental Figure 4

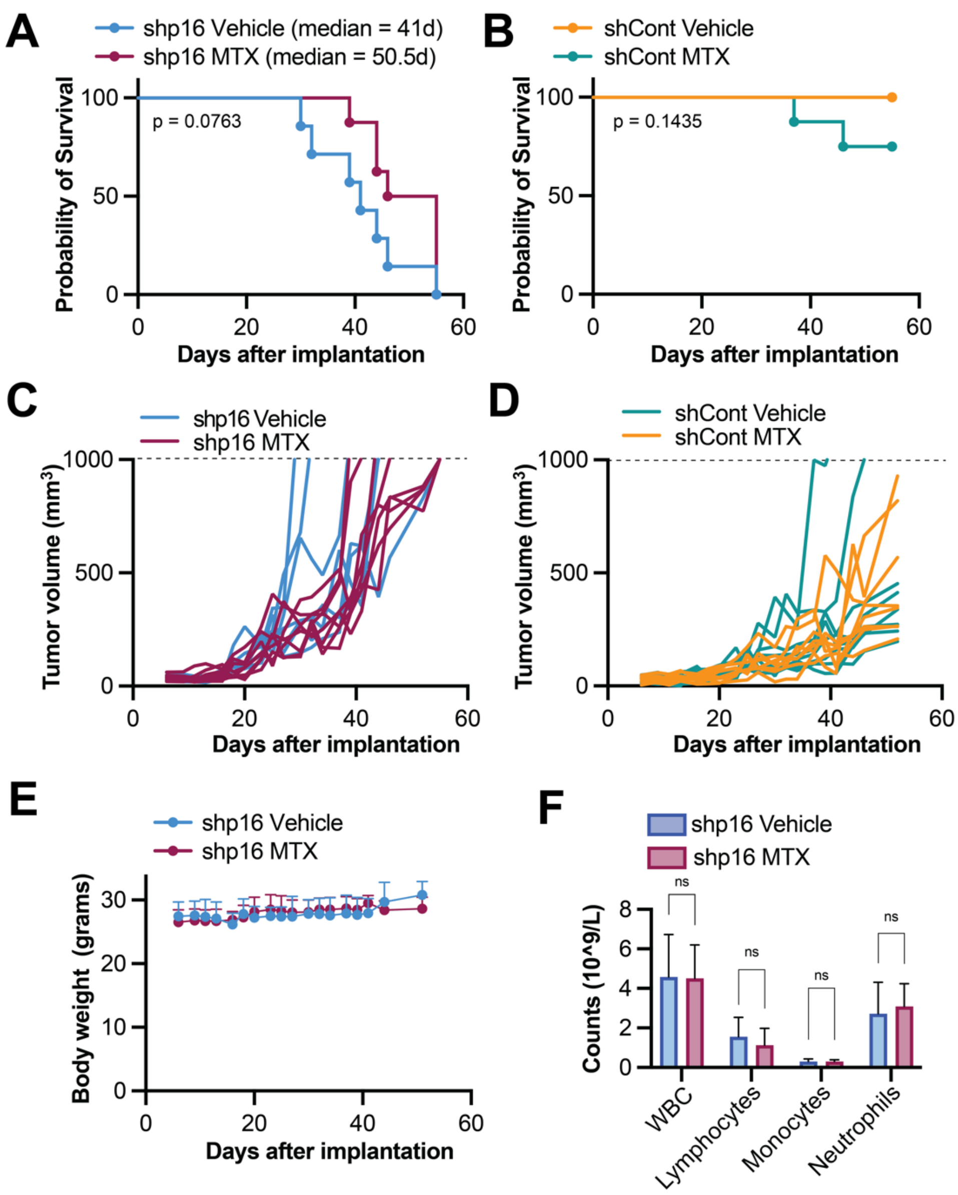

Figure S4. shp16 tumor bearing mice treated with methotrexate have a trend towards a survival advantage; and methotrexate does not affect body weight or blood cell counts. Related to Figure 5. (A-D) SKMEL28 human melanoma cells were infected with lentivirus expressing a short hairpin RNA (shRNA) targeting GFP (shCont) or p16 (shp16). 10^7 cells were subcutaneously implanted into athymic nude mice. Mice were treated with vehicle control or methotrexate (MTX). **(A-B)** Kaplan-Meier survival curves at the timepoint when all shp16 tumor bearing mice reached endpoint ($1000mm^3$). Log-rank p-values are shown. **(C-D)** Individual tumor growth curves in the indicated groups. Dotted lines indicate IACUC endpoint of $1000mm^3$ tumor volume. **(E)** Mice body weight over time. **(F)** Blood cell counts of the indicated shp16 mice at endpoint. Data are mean ± SD. T-test. ns = not significant.
